# Tumor-Associated Microglia and Macrophages in the Glioblastoma Microenvironment and Their Implications for Therapy

**DOI:** 10.3390/cancers13174255

**Published:** 2021-08-24

**Authors:** Rikke Sick Andersen, Atul Anand, Dylan Scott Lykke Harwood, Bjarne Winther Kristensen

**Affiliations:** 1 Department of Pathology, Odense University Hospital, 5000 Odense, Denmark; rikke.sick.andersen@rsyd.dk (R.S.A.); aanand@health.sdu.dk (A.A.); 2 Department of Clinical Research, University of Southern Denmark, 5000 Odense, Denmark; 3 Department of Pathology, The Bartholin Institute, Rigshospitalet, Copenhagen University Hospital, 2100 Copenhagen, Denmark; dylan.scott.lykke.harwood@regionh.dk; 4 Department of Clinical Medicine and Biotech Research and Innovation Center (BRIC), University of Copenhagen, 2200 Copenhagen, Denmark

**Keywords:** tumor-associated microglia and macrophages, TAM, glioblastoma, crosstalk, therapeutic strategies, microenvironment

## Abstract

**Simple Summary:**

Patients with glioblastoma—the most frequent and malignant primary brain tumor—have a poor prognosis with a median survival of less than 15 months. Despite extensive research, treatment of glioblastoma has not improved since 2005. Most therapeutic strategies have focused on eliminating the glioblastoma cells themselves, but tumor-infiltrating immune cells are potential targets as well. Glioblastomas are highly infiltrated with tumor-associated microglia and macrophages (TAMs), which are known to support the glioblastoma cells and promote tumor progression. Although broad categories are used to describe TAM phenotypes, recent technological advances have allowed deeper insights into their phenotypical differences. A better understanding of how known and unknown TAM phenotypes interact with their neighboring cancer cells may be crucial for establishing effective treatment strategies for glioblastoma patients. In this review, we give an updated overview of the role of TAMs in glioblastoma, and we discuss TAM–glioblastoma crosstalk and review potential therapeutic TAM-targeting strategies.

**Abstract:**

Glioblastoma is the most frequent and malignant primary brain tumor. Standard of care includes surgery followed by radiation and temozolomide chemotherapy. Despite treatment, patients have a poor prognosis with a median survival of less than 15 months. The poor prognosis is associated with an increased abundance of tumor-associated microglia and macrophages (TAMs), which are known to play a role in creating a pro-tumorigenic environment and aiding tumor progression. Most treatment strategies are directed against glioblastoma cells; however, accumulating evidence suggests targeting of TAMs as a promising therapeutic strategy. While TAMs are typically dichotomously classified as M1 and M2 phenotypes, recent studies utilizing single cell technologies have identified expression pattern differences, which is beginning to give a deeper understanding of the heterogeneous subpopulations of TAMs in glioblastomas. In this review, we evaluate the role of TAMs in the glioblastoma microenvironment and discuss how their interactions with cancer cells have an extensive impact on glioblastoma progression and treatment resistance. Finally, we summarize the effects and challenges of therapeutic strategies, which specifically aim to target TAMs.

## 1. Introduction

Glioblastoma is ranked as a grade 4 glioma by the World Health Organization (WHO) [1] and is the most frequent and malignant adult brain tumor. The median survival is less than 15 months, and 95% of patients die within 5 years of diagnosis, because an effective treatment has not yet been found [2,3]. Currently, the standard treatment for glioblastoma is surgery followed by radiation therapy along with temozolomide chemotherapy [4].

The glioblastoma microenvironment consists of several types of cells that affect the cancer cells in various ways. The tumor microenvironment (TME) primarily contains tumor-associated microglia and macrophages (TAMs), which constitute up to 30% of the total tumor [5,6,7]. TAMs secrete various factors—e.g., cytokines and growth factors—which lead to or support different biological functions in the TME, such as stemness, proliferation, angiogenesis, cancer cell migration, and immune suppression. Notably, specific subsets of TAMs are associated with poor survival in glioma patients [7,8]. Over the past years, TAMs have become an attractive target to treat glioblastoma patients, and many studies and clinical trials have focused on this.

In this article, we review and discuss the existence of TAM subtypes in the glioblastoma microenvironment based on recent cutting-edge research as well as well-established knowledge. We discuss TAM–tumor crosstalk and the extensive impact TAMs have on glioblastoma progression. Finally, we consider promising therapeutic concepts targeting TAMs.

## 2. TAMs in the Glioblastoma Microenvironment

The TME in glioblastoma consists of many different cell types. Various immune cells reside in the TME and affect the cancer cells. Myeloid cells are the most frequent cells including myeloid-derived suppressor cells (MDSCs), dendritic cells, neutrophils, in addition to TAMs, which are the most abundant in the TME [9]. The main lymphoid cells present in glioblastoma are T cells; however, they are infrequent and comprise less than 2% of the tumor [10,11] with CD8^+^ cytotoxic T cells being more frequent than CD4^+^ helper T (Th) cells and regulatory T cells (Tregs). The role of cytotoxic T cells is to eradicate abnormal cells, such as cancer cells, however, when present, cytotoxic T cells are often highly exhausted and dysfunctional, explaining why they are not as effective in glioblastoma as they are in other cancers [12,13]. Th cells assist in activating the cytotoxic T cells, whereas Tregs are immune inhibitory cells, which inhibit the cytotoxic T cell response. In addition, non-immune cells like astrocytes, neurons, endothelial cells, and pericytes are present in the TME and support the tumor in different ways [14].

As mentioned, TAMs comprise up to 30% of the entire tumor [6,7] and their presence has been associated with increased tumor growth, increased invasiveness, and inferior survival [7,15,16,17]. TAMs are, in contrast to cancer cells, genetically stable, making these cells favorable therapeutic targets. TAMs are, however, not a uniform group of cells. They comprise both microglia and macrophages [18]. The tumor-infiltrating macrophages can be blood-derived [18] or microglia-derived [19,20,21]. Previously, microglia and macrophages were distinguished by their differential expression of CD45; however, CD45 has been shown to occasionally be upregulated in microglia to the same level as in macrophages [22]. Today, a few markers have been identified for human microglia (*Tmem119* [23] *P2RY12* [24]), but no well-defined markers for human macrophages have been identified. CD49d has been suggested to distinguish macrophages [18], although this requires further validation. Microglia and macrophages may morphologically present as two distinct populations [25], but in some cases, the co-expression of microglia markers and markers frequently expressed by macrophages have been observed [26].

**Figure 1:**
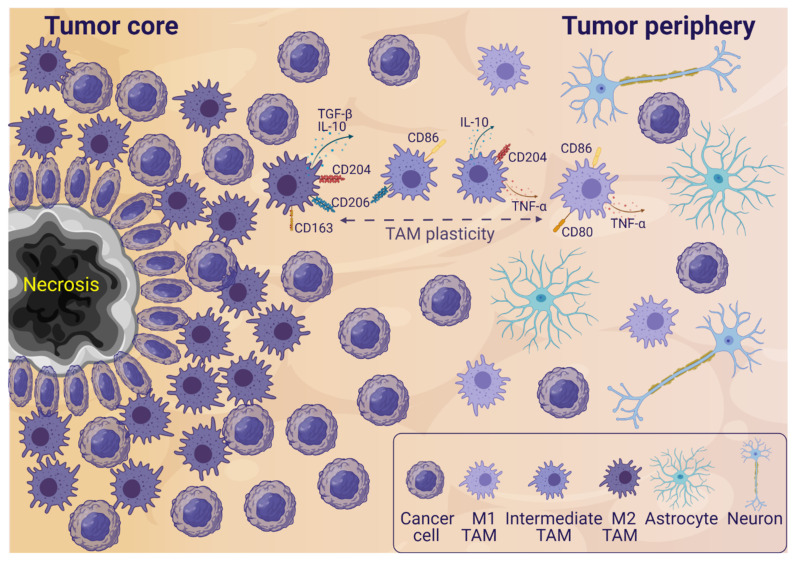
Simplified glioblastoma microenvironment illustrating a section of a tumor with both tumor core and peripheral areas. In the tumor core, a necrotic area surrounded by pseudopalisading cells is shown. Necroses surrounded by pseudopalisading cells is a glioblastoma hallmark. Anti-inflammatory M2-like tumor-associated microglia and macrophages (TAMs) are mainly infiltrating the tumor core, and pro-inflammatory M1-like TAMs are mainly infiltrating the tumor periphery. Intermediate TAMs co-expressing M1 and M2 markers are also observed in the tumor microenvironment (TME). In the periphery, there is a higher presence of non-cancer cells like astrocytes and neurons, while the tumor core is more dense with cancer cells and TAMs. More cells are present in the TME—this is a simplified illustration. Abbreviations: transforming growth factor beta (TGF-β), interleukin (IL), tumor necrosis factor alpha (TNF-α). Created with BioRender.com (accessed on 16 August 2021).

Historically, two main TAM phenotypes have been described: the classical, pro-inflammatory M1 TAMs and the alternatively activated, anti-inflammatory M2 TAMs. M2 macrophages have been divided into three subtypes—M2a, M2b, and M2c—with very different functions, such as the involvement in allergy, immune regulation, and tissue remodeling [9]. Simplistically, M1 TAMs are anti-tumorigenic, secreting pro-inflammatory cytokines, like tumor necrosis factor-α (TNF-α), and mediating Th1 responses. M2 TAMs have pro-tumorigenic functions and secrete anti-inflammatory cytokines—such as transforming growth factor-β (TGF-β)—which inhibit cytotoxic T cells and attract Tregs and MDSCs. Furthermore, they produce factors supporting extracellular matrix remodeling as well as angiogenesis [9,27]. However, these two main TAM phenotypes have been revealed to be the ends of a spectrum, and TAMs are now considered as cells with high plasticity, which can assume many functions and phenotypes [28]. In addition, heterogeneous populations of TAMs co-expressing M1 and M2 markers have been identified in both glioblastoma [7,25,29,30] as well as a model of CNS injury [31], which illustrates the presence of differential phenotypes, as well as phenotype switching (Figure 1). Moreover, an unforeseen myeloid cell heterogeneity was observed in an orthotopic glioma mouse model, underlining the substantial heterogeneity of these cells [32].

A proper characterization of the functional and phenotypical heterogeneity of TAMs requires future studies to investigate the spatial context of these cells, and how they interact with neighboring cells. These studies are beginning to emerge, with the very recent identification of a subpopulation of *HMOX1*-expressing TAMs that drive T cell exhaustion [33]. This study showed a correlation between T cell exhaustion and *HMOX1*-expressing myeloid cells in a spatial context. Another recent study identifies six different subsets of TAMs with distinct gene signatures that associate with, e.g., hypoxia, phagocytosis or an interferon-induced signature [34]. In addition, this study associates TAMs with T cell suppression and angiogenesis.

Most studies describe TAMs in the tumor core. However, there is high histological heterogeneity in glioblastomas, with the presence of different histological structures, such as tumor core, pseudopalisading cells around necrosis, microvascular proliferation and tumor periphery [35]. TAM presence varies between the different structures, with the highest frequency in the tumor core and around necrotic areas with hypoxia [36,37] (Figure 1). It has been shown that TAMs in the tumor core are mainly macrophages with an anti-inflammatory, M2-like profile, while TAMs in the periphery are mainly microglia with a more pro-inflammatory, M1-like profile [25,38].

Studies associate different TAM populations with poor prognosis. Several studies have associated infiltration of both M2-like microglia and macrophages with inferior survival [7,39,40] as well as resistance to radiotherapy [16] in high-grade gliomas. One of the studies showed that the presence of M2-like, CD204^+^ TAMs is an independent predictor of survival in glioblastomas [7]. In other studies, only macrophages display the anti-inflammatory and pro-angiogenic phenotype, while microglia are classified as pro-inflammatory [25,38]. These studies suggest that only macrophages and not microglia are pro-tumorigenic. In addition, it has been proposed that the presence of macrophages in low-grade gliomas correlates with inferior survival [25], and they are more abundant in higher-grade gliomas than microglia [26]. The therapeutic targeting of pro-tumorigenic TAMs is highly favorable and is of great focus in the search to improve the survival of glioblastoma patients.

The glioblastoma TME is complex, with tumor heterogeneity being a large contribution to complexity. Different molecular subtypes exist: proneural, mesenchymal, and classical, and these are associated with copy number amplification in *PDGFRA*, inactivating mutations in *NF1,* and copy number amplification in *EGFR*, respectively [16,41]. These subtypes can either be the main characteristic of an individual tumor—e.g., a mainly mesenchymal tumor—or the different subtypes can be present in different regions within the same tumor [42,43]. TAMs are most abundant in the mesenchymal subtype, which generally has the highest degree of infiltrating immune cells [11,44]. *NF1* deficiency is a characteristic of malignant cells of the mesenchymal subtype and has also been linked to increased chemoattraction and infiltration of TAMs [16].

## 3. Glioblastoma and TAM Crosstalk

Glioblastoma cells secrete several factors that regulate TAM phenotype, survival and recruitment to the TME. After recruitment to the tumor, TAMs support and promote glioblastoma cell migration, invasion, stemness, proliferation, angiogenesis and immune suppression. Below we discuss the latest and most well established knowledge on these aspects, first with a focus on glioblastoma-derived factors and next on TAM-derived factors.

### 3.1. The Influence of Glioblastoma Cells on TAMs

**Figure 2:**
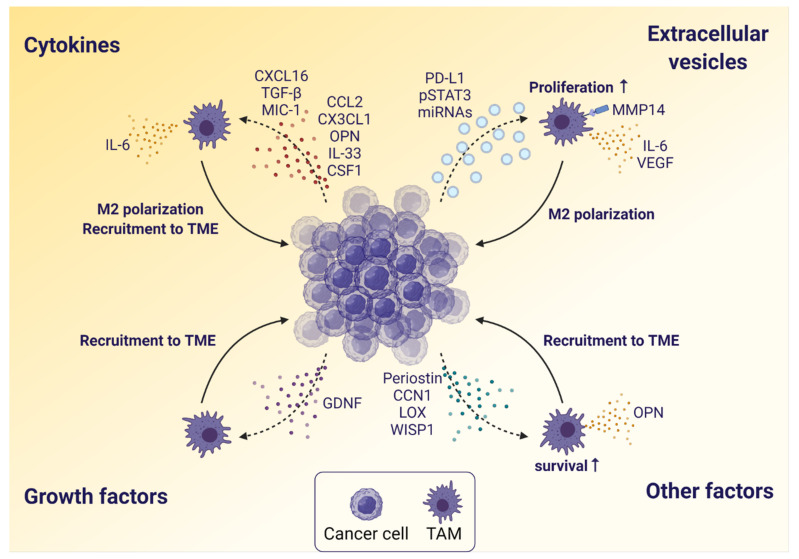
The influence of glioblastoma cells on TAMs. Glioblastoma cells secrete various factors that affect TAMs in different ways. Secreted cytokines lead to recruitment of TAMs to the TME, and to polarization of TAMs towards the pro-tumorigenic M2-like phenotype. In addition, TAMs are stimulated to secrete cytokines that further enhance recruitment and tumor invasion. Secreted growth factors also lead to recruitment of TAMs. Glioblastoma cells can also release extracellular vesicles, which are taken up by TAMs and affect their phenotype by induction of M2-like polarization and expression of matrix metalloproteinase 14 (MMP14), IL-6 and vascular endothelial growth factor (VEGF), contributing to a more progressive tumor. In addition, TAM proliferation may be increased. Other factors are secreted as well, leading to recruitment of TAMs to the TME as well as to stimulation of TAM survival and cytokine secretion. Abbreviations: C-X-C motif chemokine ligand (CXCL) 16 (CXCL16), macrophage inhibitory cytokine-1 (MIC-1), C-C motif chemokine ligand (CCL), C-X3-C motif chemokine ligand 1 (CX3CL1), osteopontin (OPN), colony-stimulating factor 1 (CSF1), glial cell-derived neurotrophic factor (GDNF), cellular communication network factor 1 (CCN1), lysyl oxidase (LOX), Wnt-induced signaling protein 1 (WISP1), programmed death ligand 1 (PD-L1), microRNAs (miRNAs). Created with BioRender.com (accessed on 5 July 2021).

Several factors are involved in the crosstalk between glioblastoma cells and TAMs (Figure 2). Most of these factors either attract and recruit TAMs to the tumor or polarize the TAMs towards a more pro-tumorigenic M2-like phenotype. Thus, the tumor modifies and exploits the TAMs to support its malignant progression. The majority of these known glioblastoma-secreted factors are cytokines, and many of these function as chemo-attractants, such as C-X3-C motif chemokine (CX3C) ligand 1 (CX3CL1), C-C motif chemokine ligand (CCL) 2 (CCL2), macrophage inhibitory cytokine-1 (MIC-1), and colony-stimulating factor 1 (CSF1) [45,46,47]. The chemokine CX3CL1 is involved in the recruitment of TAMs to the glioblastoma TME via tumor secretion and subsequent binding to CX3C receptor 1 (CX3CR1) on TAMs [46,48].

Moreover, C-X-C motif chemokine ligand (CXCL) 16 (CXCL16) is secreted by glioblastoma cells, and by signaling through C-X-C motif chemokine receptor (CXCR) 6 (CXCR6) it polarizes TAMs towards the M2-like phenotype. CXCL16/CXCR6 signaling can also act directly on tumor cells to promote their proliferation, migration and invasion in both human glioblastoma cells in vitro as well as in a glioma mouse model [47]. IL-33 secreted by glioblastoma cells is involved in TAM recruitment and polarization towards pro-tumorigenic M2-like TAMs. In addition, IL-33 expression is associated with poor survival in glioblastoma patients [49]. The cytokine osteopontin (OPN) is secreted by glioblastoma cells, and it may affect these in a variety of ways, such as induction of invasion, survival and angiogenesis, but it also recruits macrophages to the TME via integrins and maintains the M2-like phenotype [50,51]. Additionally, glioma-derived CCL2 recruits TAMs to the tumor [52], but at the same time takes part in a feed-forward mechanism, where it induces IL-6 production in microglia, which consecutively affects glioma cells by increasing their invasiveness [53]. TGF-β is a multifunctional cytokine, which is secreted by both TAMs and glioblastoma cells, and it promotes various processes in the tumor, like cancer cell proliferation, invasion, stemness, angiogenesis and immune suppression, but TGF-β also recruits TAMs to the TME and plays a role in M2-like polarization [45,54].

In addition to cytokines, glioma cells also produce the growth factor glial cell–derived neurotrophic factor (GDNF), which plays a critical role in controlling TAM recruitment to the TME through GDNF family receptor alpha 1 and 2 expressed by TAMs. Interestingly, depleting GDNF in a glioma cell line and implanting the cells in mouse brains shows reduced tumor size and prolonged survival of the mice [55].

Glioblastoma cells also produce additional factors. For instance, the cell cycle regulator cellular communication network factor 1 (CCN1) plays an important role in cancer progression and inflammatory response in different solid cancers [56,57]. In glioblastoma, secretion of CCN1 is reported to result in macrophage infiltration in an in vivo model through the upregulation of integrins [58]. Another glioblastoma-derived factor influencing TAMs is periostin, which is secreted to recruit TAMs to the tumor. Periostin expression correlates with TAM density, and depletion of periostin in glioblastoma cells reduces TAM recruitment and inhibits tumor growth in mice bearing glioma stem-like cell-derived xenografts via integrin αvβ3 signaling [59]. Furthermore, *PTEN* deficient glioma cells have been shown to secrete lysyl oxidase (LOX), which recruits macrophages to the tumor. In a *PTEN*-null glioblastoma xenograft mouse model, LOX inhibition decreases macrophage infiltration and tumor growth [51]. LOX-mediated macrophage recruitment triggers the secretion of OPN by macrophages, which further recruits additional macrophages. Recently, Wnt-induced signaling protein 1 (WISP1) was reported to be secreted by glioblastoma stem-like cells and to promote the survival of both glioblastoma cells and TAMs by Akt signaling. Targeting WISP1 signaling decreases the tumor and TAM load in glioblastoma mouse models [60].

Finally, extracellular vesicles released by glioblastoma cells play a critical role in tumor progression [61]. Glioblastoma cells release extracellular vesicles containing programmed death (PD) ligand 1 (PD-L1) and phospho-STAT3, which can be taken up by TAMs and polarize them towards the M2-like phenotype [62]. Additionally, extracellular vesicles from glioblastoma cells are also known to influence primary human microglia by increasing their expression of matrix metalloproteinase 14 (MMP14), vascular endothelial growth factor (VEGF), and IL-6, thereby making them more pro-tumorigenic [63]. Similarly, glioma-derived exosomes can carry microRNAs (miRNAs) that after uptake by TAMs can affect TAM phenotype and functions. Of these, miR-1246 has been shown to induce M2 polarization, which involves the inhibition of the NF-κB pathway as well as activation of the STAT3 pathway [64]. The oncogenic miR-21 has been shown to increase microglial proliferation [65].

Some tumor-derived factors influencing TAMs have been therapeutically targeted in clinical trials. E.g., CCL2 was inhibited to reduce TAM recruitment to the tumor, and CD47 has been targeted to reduce its inhibitory effect on TAM phagocytosis. These will be described further in the following section.

### 3.2. The Influence of TAMs on Glioblastoma Cells

**Figure 3:**
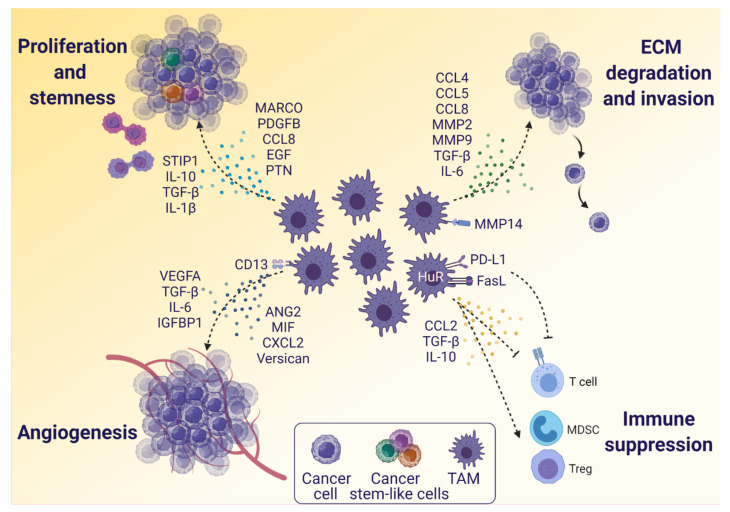
The influence of TAMs on glioblastoma cells. TAMs express many factors that promote major biological functions in tumor progression. Glioblastoma proliferation and stemness are highly affected by TAM-secreted factors, such as cytokines and growth factors. Angiogenesis, the essential process of neovascularization in glioblastoma tumors, is also promoted by several TAM-derived factors, including VEGF and VEGF-inducing factors, but also VEGF-independent pathways. Additionally, glioblastoma invasion is promoted by TAM-produced MMPs and cytokines inducing extracellular matrix (ECM) degradation and facilitating migration. Moreover, TAMs express factors to suppress anti-tumor immune responses. Pro-inflammatory cytotoxic T cells and helper T cells are inhibited, while anti-inflammatory myeloid-derived suppressor cells (MDSCs) and regulatory T cells (Tregs) are attracted to the tumor, where they facilitate tumor immune evasion. Abbreviations: stress-induced phosphoprotein 1 (STIP1), macrophage receptor with collagenous structure (MARCO), platelet derived growth factor B (PDGFB), epidermal growth factor (EGF), pleiotrophin (PTN), insulin-like growth factor-binding protein 1 (IGFBP1), angiopoietin (ANG2), macrophage migration inhibitory factor (MIF), Fas ligand (FasL). Created with BioRender.com (accessed on 16 August 2021).

After their recruitment to the glioblastoma TME, many TAMs differentiate into the M2-like phenotype. High expression of M2-like TAM markers—e.g., CD204 and CD163—in glioblastoma are associated with poor survival and an aggressive glioma subtype [7,66]. TAMs produce and secrete various factors—including cytokines and growth factors—which control different biological functions in the TME, such as proliferation, stemness, angiogenesis, migration, invasion, and immune suppression. Below, we discuss TAM effects on glioblastoma cells (Figure 3).

Sustained cell proliferation and stem cell-like characteristics are important factors in glioblastoma biology and sustained proliferative signaling is a hallmark of glioblastoma [67,68]. Glioblastoma stem-like cells are a critical factor in glioblastoma progression and relapse [69,70]. They have been described to reinitiate tumor growth after the removal of therapeutic pressure, leading to tumor recurrence [71,72]. Many signaling factors released by TAMs play critical roles in glioblastoma stemness and proliferation. Epidermal growth factor (EGF) receptors (EGFRs) play crucial roles in glioma cell proliferation, and tumor-associated microglia secrete EGF that activates EGFR in glioblastoma cells [73,74,75]. In addition, stress-induced phosphoprotein 1 (STIP1) secreted by TAMs induces proliferation of glioma cells in vitro [76,77], while IL-10 was shown to promote glioma cell proliferation via JAK2/STAT3 signaling in a glioma model [78]. Recent results show that TAMs secrete IL-1β, which promotes the rate of glycolysis in glioma cells through glycerol-3-phosphate dehydrogenase (GPD) enzyme. Hindering IL-1β signaling in TAMs attenuated the glycolysis rate and cell proliferation of glioma cells in vitro [79]. The same study showed a positive association between the activation of GPD and IL-1β expression, TAM recruitment, tumor grade, and reduced survival in human glioblastoma patients. TGF-β also increases the proliferation of glioblastoma cells. Under normal circumstances, TGF-β has cytostatic effects, but in cancer TGF-β signals via Smad4, which induces expression of platelet derived growth factor B (PDGFB) and thereby glioma proliferation [80]. The multifunctional TGF-β can also enhance stemness in glioma cells [80,81]. Moreover, a glioblastoma study shows that TAMs overexpress macrophage receptor with collagenous structure (MARCO), which drives a phenotypic shift to the mesenchymal subtype, as well as induce invasiveness, proliferation and therapeutic resistance [82]. CCL8 and hypoxia-induced CCL4 signals also induce an invasive phenotype and glioma stem-like traits in glioblastoma cells [83,84]. Additionally, TAMs are also known to secrete pleiotrophin (PTN) that promotes glioblastoma tumor growth and stem cell maintenance via protein tyrosine phosphatase receptor type Z1 (PTPRZ1) signaling [85]. Although the above-mentioned factors play crucial roles in stemness and glioma cell proliferation, detailed mechanisms and further knowledge is required from fresh human glioblastoma tissue.

Cancer cells require a continuous supply of oxygen and nutrients, and angiogenesis is part of the biological program to support this. After recruitment to the TME, TAMs release different factors—such as VEGF, IL-6, Angiopoietin 2 (ANG2), and insulin-like growth factor-binding protein 1 (IGFBP1)—to stimulate neovascularization in the tumor [86,87,88,89]. The angiogenic role of VEGF-A to stimulate endothelial ECM degradation, proliferation, and migration to form new blood vessels in tumors is well established, and it is an important mediator of hypoxia-induced tumor growth in glioblastoma [88]. VEGF-A is both tumor- and TAM-derived. It has been shown that transgenic mice with VEGF-A-deficient myeloid cells implanted with glioma cells have delayed tumor growth and prolonged survival, indicating that TAM-derived VEGF-A plays a significant role in tumor progression [90]. However, other factors play essential roles in glioblastoma angiogenesis as well, which is supported by the fact that anti-VEGF-A treatment with Bevacizumab is ineffective [91,92]. The multifunctional cytokine TGF-β can act as an angiogenic factor by upregulating VEGF as well as the plasminogen activator inhibitor (PAI-I), which is involved in vessel maturation [93]. Recently, high levels of macrophage migration inhibitory factor (MIF) have been associated with tumor recurrence and poor survival, and MIF is now emerging as a promising anti-angiogenic target in glioblastoma [94,95,96,97]. Hypoxia-induced MIF has been shown to be released in the glioblastoma TME promoting vasculogenic structure, and its expression correlates with the expression of VEGF [98,99]. Furthermore, microglia produce IGFBP1 in response to glioma-derived CSF1, and IGFBP1 has been shown to induce angiogenesis in glioma cells [89]. Versican is a proteoglycan that has various pro-tumorigenic properties. It was revealed to increase the survival and angiogenic properties of glioblastoma cells in vitro and in vivo in mice [100,101]. Alternative proangiogenic factors were recently suggested to be expressed by TAMs infiltrating glioblastomas having high granulocyte infiltration. These TAMs showed high expression of CD13 and CXCL2, which may promote angiogenesis independent of VEGF [102,103].

Extracellular matrix (ECM) in glioblastoma is an important structural factor that contains mainly proteoglycans and glycosaminoglycans [104]. TAM-derived factors—such as CCL8, CCL5, and MMPs—play crucial roles in tumor invasion and progression by participating in ECM degradation. A mouse glioblastoma study showed that CCL8, primarily secreted by TAMs, helps in the formation of pseudopodia-like structures in glioblastoma cells via C-C motif chemokine receptor (CCR) 1 (CCR1) or CCR5 axes, which promotes tumor migration and invasion. Additionally, CCL8 activates the ERK1/2 pathway that also leads to invasion as well as increased stemness of glioblastoma cells. Targeting glioma cells with an ERK1/2 inhibitor leads to decreased invasion [83]. Additionally, microglia-derived CCL5 triggers upregulation of MMP2 in glioma cells through calcium and Akt signaling, which in response promotes ECM degradation and invasiveness in glioma [105]. Similarly, MMP2 can also be expressed by microglia themselves [106]. Moreover, M2-like macrophages express MMP9 and MMP14, which are also associated with ECM degradation and facilitate cancer cell migration through the TME [107,108,109,110]. Microglia also secrete TGF-β, which has been shown to have an invasion-promoting role in glioblastoma cells both in vitro and in vivo in mice [111]. The effect of microglia-derived TGF-β may be mediated by upregulated MMPs and downregulated tissue inhibitor of metalloproteinases (TIMP) [112]. As mentioned earlier, glioma cells secrete CCL2, which induces IL-6 secretion in microglia thereby increasing glioma invasiveness [53]. The enhanced invasion may be mediated by an IL-6-induced increase in STAT3 signaling and upregulation of MMP2 in glioma cells [113].

Apart from promoting cancer cells, TAMs also play crucial roles in T cell inactivation and immune suppression leading to tumor immune evasion. M2-like TAMs express PD-L1 that may lead to T cell inactivation. Treating a glioblastoma mouse model with anti-PD-L1 antibody shows reduced infiltration of M2-like TAMs and reduced tumor growth [114,115]. Notably, monocytes from glioblastoma patients have elevated levels of PD-L1 compared to healthy individuals, and co-culturing PD-L1^+^ monocytes with T cells leads to T cell apoptosis [116]. Additionally, a glioblastoma mouse study indicates that anti–PD-L1 treatment may boost a radiotherapy response by mediating activation of T cells and macrophages [117]. The Fas ligand (FasL) is also well known for its role in immune suppression by inducing T cell apoptosis. Evidence from a mouse glioma model suggests that microglia are a major source of FasL in gliomas and inhibiting FasL elevates the leukocyte infiltration in tumors [118,119]. In addition, TAMs secrete CCL2, which is critical for the recruitment of Tregs and MDSCs to the tumor. High expression of CCL2 in glioblastoma patients is associated with poor survival [120]. Additionally, M2-like TAMs secrete IL-10 and TGF-β that play critical roles in tumor immune evasion by inhibiting Th1 and cytotoxic T cell effector molecules, inhibiting antigen presentation by downregulating MHC class II on glioma cells, and attracting immune-suppressive Tregs and MDSCs [27,111,121,122,123,124].

The RNA regulator human antigen R (HuR) complex post-transcriptionally regulates several mRNAs in the STAT and NF-kB signaling pathways, and it plays a role in shaping the TAM molecular signature and phenotype. The deletion of the HuR gene in TAMs leads to the accumulation of pro-inflammatory M1-like TAMs and cytotoxic T cells in the TME, which further causes tumor suppression and prolonged survival in a HuR knockdown mouse model of glioblastoma [125,126].

As illustrated in this section, extensive crosstalk between TAMs and cancer cells occurs in the glioblastoma TME. Many attempts have been made to target TAM–glioblastoma interactions for therapeutic intervention in clinical trials, and these are subject for discussion in the next section.

## 4. Glioblastoma Treatment—Therapies Targeting TAMs and Challenges

For more than a decade, only limited advancement in the treatment of glioblastoma has been achieved despite extensive efforts to develop new therapeutic strategies. TAM-targeting strategies have been and are being clinically tested. TAMs may be responsible for part of glioblastoma treatment resistance. Radiation therapy leads to TAM recruitment to the tumor. Especially the numbers of macrophages are increased in the tumor [127,128], and upon recurrence, there is a higher macrophage-to-microglia ratio [129]. Furthermore, radiotherapy induces a more M2-skewed TME, which might be explained by increased radio-resistance of M2-like TAMs, as suggested by a preclinical glioblastoma study [130]. One study found TAMs to be involved in the radio-resistance of glioblastoma cells by the secretion of TNF-α, which increases nuclear factor-κB (NF-κB) signaling and induces the radio-resilient mesenchymal subtype [131]. The study also found that the mesenchymal gene signature, CD44 expression and NF-κB activation correlated with inferior survival and poor radiation response.

**Figure 4:**
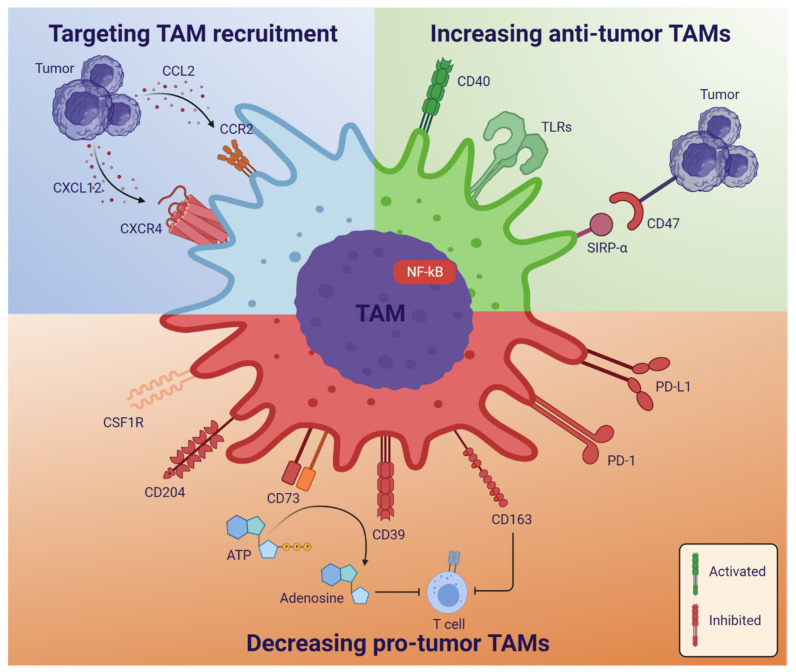
Therapeutic TAM-targeting in the treatment of glioblastoma. TAMs are potential therapeutic targets in the fight against glioblastoma, and these can be targeted in different ways. The recruitment of TAMs to the tumor can be targeted by inhibiting the chemokine signaling that attract TAMs to the TME (see the blue TAM part). A second strategy is to increase the numbers of anti-tumor M1-like TAMs to improve anti-tumor immune responses (see the green TAM part). A third strategy is to decrease the numbers of pro-tumor M2-like TAMs, which could in turn improve anti-tumor immune responses and possibly inhibit tumor progression (see the red TAM part). Targets illustrated in green should be activated by therapeutic treatments, while targets in red should be inhibited, as illustrated by the key in the bottom corner. Abbreviations: C-C motif chemokine receptor 2 (CCR2), C-X-C motif chemokine receptor 4 (CXCR4), CSF1 receptor (CSF1R), programmed death-1 (PD-1), signal regulatory protein-α (SIRP-α), toll-like receptors (TLRs). Created with BioRender.com (accessed on 5 July 2021).

TAMs can be therapeutically targeted in different ways: (1) targeting TAM recruitment to the tumor, (2) reprogramming TAM polarization towards a more anti-tumor, M1-like phenotype, or (3) decreasing or eliminating tumor-promoting M2-like TAMs (Table 1 and Figure 4).

### 4.1. Targeting TAM Recruitment

TAM recruitment can be prevented by inhibiting the chemokine gradient axes involved in recruiting TAMs to the tumor, such as the CCL2-CCR2 and CXCL12-CXCR4 axes. The inflammatory chemokines CCL2 and CXCL12 attract CCR2- and CXCR4-expressing monocytes, microglia, and macrophages. When secreted by cancer cells, monocytes, microglia and macrophages are recruited to the tumor, contributing to an immunosuppressive TME [52,155].

Anti-CCL2 monoclonal antibodies (mAbs) and small molecule inhibitors of CCR2 have been tested in phase I/II clinical trials in solid tumors [132,133,134]. These treatments are generally well-tolerated, although only CCR2 inhibition combined with chemotherapy obtained objective responses and awaits further investigation in randomized trials [134].

A CXCR4 antagonist has been investigated in glioblastoma patients. In combination with Bevacizumab (anti-VEGF), it showed no effect on survival [136], but in combination with chemoradiation (temozolomide), median overall survival was improved to 21.3 months [135], compared to 14.6 months for chemoradiation alone [156]. This is being investigated further in a follow-up trial (NCT03746080).

### 4.2. Reprogramming TAM Polarization and Increasing Anti-Tumor TAMs

Numerous studies aim at increasing the M1/M2 TAM ratio to polarize TAMs towards the pro-inflammatory, anti-tumorigenic M1-like phenotype, and several targets are being investigated—also in the clinic.

CD47 is a checkpoint molecule inhibiting macrophage phagocytosis upon binding to the signal-regulatory protein-α (SIRPα), and high expression correlates with poor survival in glioblastoma [157]. Inhibition of CD47 leads to increased phagocytosis of cancer cells by TAMs in experimental glioblastoma models, as well as an increase in M1-like macrophages and improved survival in glioblastoma animal models [158,159,160,161]. Furthermore, recent preclinical studies have shown that a combination of CD47-SIRPα axis-targeting and autophagy inhibitors may increase the anti-tumor effects of CD47 inhibition, which may in addition increase the effect of anti-PD-1 treatment [162,163]. Small molecule inhibitors of CD47 and anti-CD47 mAbs are being tested in the clinic in both solid and hematological cancers, and results are pending [137].

Toll-like receptors (TLRs) are expressed by macrophages and activation with different TLR ligands polarizes TAMs to an M1-like phenotype [164]. TLR agonists have been tested in clinical trials in both solid tumors and lymphomas, although showing little efficacy [138,139]. The effects in gliomas are currently being investigated in a few clinical trials, however, only as immune adjuvants for immunotherapies (NCT03392545, NCT01204684).

CD40 is a costimulatory molecule belonging to the TNF receptor superfamily. It is required for the activation of antigen-presenting cells and can polarize TAMs towards a more anti-tumorigenic phenotype. Many cancer cells also express CD40, and high CD40 correlates negatively with overall survival in both low- and high-grade gliomas [165]. A preclinical study showed that the combination of an anti-CD40 antibody and inhibition of CSF1 receptor (CSF1R) signaling reprogrammed TAMs to a more pro-inflammatory phenotype [166]. This combination is now being tested in a phase I clinical trial in solid cancers (NCT02760797). In recurrent glioma, an anti-CD40 antibody is being tested in combination with anti-PD-L1 and D2C7-IT—an immunotoxin conjugated to an anti-EGFR antibody fragment (NCT04547777). In addition, an anti-CD40 antibody is being tested in pediatric CNS tumors (NCT03389802).

NF-κB is a versatile transcription factor, which participates in regulating the inflammatory response. Under normal conditions, NF-κB has been described to support polarization towards M1-like macrophages [167]; however, in a cancer setting, NF-κB signaling in tumor-associated macrophages was shown to inhibit M1 polarization while M2 anti-inflammatory responses were favored [168,169]. In addition, glioblastoma growth has been shown to require myeloid NF-κB signaling [140]. Moreover, inhibition of NF-κB signaling in myeloid cells leads to M1-like TAM polarization in mouse models of glioblastoma, prostate and pancreatic cancer, which leads to increased infiltration of cytotoxic T cells and decreased tumor growth [140,141,142]. Thus, the NF-κB pathway is a potential therapeutic target in different cancers—including glioblastoma—however, more research is needed, and it is yet to be tested in the clinic.

### 4.3. Decreasing Tumor-Promoting TAMs

As discussed earlier, tumor-promoting TAMs can be either all M2-like TAMs—including both M2-polarized microglia and macrophages—or macrophages only, since some studies suggest that mainly macrophages are pro-tumorigenic, while microglia are more anti-tumorigenic [25,38]. Most research has focused on targeting the M2-like TAMs since a validated marker for macrophages still awaits. In this regard, it has been speculated to target CD49d, however, more research is necessary to proceed along this line [129,170].

CSF1 is released by cancer cells and attracts TAMs, while at the same time polarizes them towards the M2-like phenotype. CSF1R expressed by TAMs is, thus, a target for the depletion of pro-tumorigenic TAMs. CSF1R inhibitors have been tested in numerous clinical trials in solid tumors and acute myeloid leukemia [171], with the best effect in tenosynovial giant cell tumors, for which it has been FDA approved [143]. Experimentally, there is evidence of anti-tumor effects in glioblastoma models [145], but in the clinic, there is no effect of CSF1R inhibition in glioblastoma [144]. However, in glioma mouse models, CSF1R inhibitors were shown to sensitize glioma cells to tyrosine kinase inhibitors [172]. Furthermore, two recent studies using glioma and glioblastoma mouse models showed that CSF1R inhibition in combination with radiation prevents the radiation-induced M2-like polarization of TAMs and increases the response to radiation by delaying or preventing recurrent disease [129,146]. CSF1R inhibitors are also now being tested in clinical trials in combination with standard of care radiation and temozolomide (NCT01790503), as well as in combination with an anti-PD-1 antibody (NCT02829723).

The PD-1/PD-L1 checkpoint axis has been targeted in many cancers after the great success in malignant melanoma. It primarily aims to remove the brake on the anti-tumor cytotoxic T-cell response; however, T cells are scarce and often exhausted in glioblastoma [12]. Both M1- and M2-like TAMs express PD-1 and PD-L1, although M2-like TAMs express them to a higher degree [115,116,173]. PD-1 expression has been shown to correlate negatively with TAM phagocytic capacity against cancer cells [173], and PD-L1 expression may cause inhibition of T cells [116]. In mouse studies of colon cancer and glioma, PD-1/PD-L1 blockade resulted in a decrease in M2-like TAMs, increased TAM phagocytosis, reduced tumor growth and increased survival, independent of T cell presence [173,174]. Furthermore, PD-1 expression in M2-like TAMs increases with increasing disease stage in patients with colorectal cancer as well as over time in mouse models of colon cancer [173]. The clinical effect of PD-1/PD-L1 blockade in glioblastoma has not been as successful as in other cancers; however, there may be a survival benefit in a neoadjuvant compared to an adjuvant setting using anti-PD-1 in recurrent glioblastoma [147]. A phase 3 clinical trial in recurrent glioblastoma comparing anti-PD-1 with anti-VEGF (Bevacizumab) showed no difference in overall survival, but the anti-PD-1 treated group had more durable responses. In addition, a subgroup of patients with methylated MGMT promoter and no corticosteroid use had increased overall survival [148]. These studies indicate that anti-PD-1 might work in the right setting, and it is now being tested in several clinical trials in combination with other therapies [149].

One of these combination therapies could be targeting CD73, an ectonucleotidase that together with CD39 converts extracellular ATP into the anti-inflammatory factor adenosine. CD73 is expressed by a subset of TAMs with an immunosuppressive and macrophage-like gene signature [150]. Using single-cell RNA-seq, a CD73^high^ gene signature was derived, and high expression of this signature correlated negatively with overall survival in The Cancer Genome Atlas (TCGA) cohort of glioblastoma patients. In addition, these cells persisted after immune checkpoint therapy [150]. In a glioblastoma mouse model, CD73^-/-^ mice showed improved survival when treated with anti-PD-1 and anti-CTLA-4 compared to untreated mice as well as treated wild-type mice [150]. Depletion of CD73-expressing TAMs could be a potential novel treatment strategy; however, it has not been tested in glioblastoma patients. It is currently being tested in phase I and II clinical trials in several solid cancers, both alone and in combination with immune checkpoint inhibitors [151].

Like CD73, CD39 is expressed in tumor-associated macrophages, and CD39 inhibition increases the secretion of inflammatory cytokines [175]. In gliomas, CD39 expression increases with increasing disease grade, and it contributes to T cell dysfunction [152]. An anti-CD39 antibody is being tested in a phase I clinical trial in advanced solid tumors (NCT04336098) but has not been tested in glioblastomas.

CD163 is a marker for pro-tumorigenic M2-like TAMs. CD163 expression is positively associated with glioma malignancy grade, and correlates significantly with poor survival in glioblastoma as well as gliomas of all grades [66]. CD163^+^ TAMs have been shown to impair T cell function in a melanoma mouse model, while depletion of the CD163^+^ cells resulted in the infiltration of activated T cells and inflammatory monocytes, which led to tumor regression [153]. In addition, CD163^+^ TAMs isolated from human glioma patients and co-cultured with autologous peripheral T cells decreased the frequency of effector T cells in culture. Subsequent M1-polarization of the TAMs resulted in an increase in effector T cells [66]. Thus, CD163 could be an attractive therapeutic target for depletion of M2-like TAMs. It has, however, not been tested in the clinic.

Another potential new target in glioblastoma is CD204. It is also a marker for M2-like TAMs, and CD204 expression is associated with shorter survival in high-grade gliomas. In addition, the amount of CD204^+^ TAMs increases with malignancy grade, and CD204 has been shown to be of independent prognostic value in glioblastoma [7,154]. Currently, the targeting of CD204 has not been tested in clinical trials, but it may be an attractive therapeutic opportunity.

### 4.4. Combination Therapies and Translational Challenges

Many TAM-targeting therapies are being tested as monotherapies; however, there could be an advantage in combining different treatments. It seems that combining radiation or chemoradiation with TAM targeting—such as inhibition of CXCR4 or CSF1R—can repolarize or deplete the pro-tumorigenic TAMs that infiltrate the tumor after radiation, improving the radiation effect [129,135,146]. In addition, PD1 targeting could have a better effect if combined with targeting of, e.g., CD73, which inhibits cytotoxic T cell function [150].

Selecting patients based on molecular subtypes or gene expression could also aid in getting a better response to a treatment [176]. For example, in the before-mentioned phase 3 trial with anti-PD-1 treatment, the patients with methylated MGMT promoter had improved survival compared to patients with non-methylated MGMT promoter [148].

Often, preclinical experiments look promising for new targets and therapies; however, there is a poor correlation between existing in vitro and in vivo models and human glioblastoma. In vitro models using cell lines, glioma spheres, tumor organoids or patient-derived cells lack the complete picture of the tumor, both with regard to tumor heterogeneity and subtype, as well as inclusion of the complete TME. In vivo models can give a more complete biological context. Xenograft models—such as the syngeneic, orthotopic GL261 glioma mouse model—have contributed considerably to the understanding of glioma biology; however, even similar experiments investigating anti-PD-1 checkpoint blockade in the GL261 model have shown huge variations [177]. Patient-derived xenografts are also used, and they retain the genetic and histological features of the primary tumor; however, the drawback is that the mouse is often immune deficient to avoid tumor rejection. Today, complex mouse models with humanized immune systems have been made by transplantation of human peripheral blood lymphocytes and/or CD34^+^ hematopoietic stem cells into immune-deficient mice [178]. These models could potentially contemplate the TME better, but there are still issues to be solved, such as host innate immune responses, incomplete human immune function and limited lifespan [179]. In addition, their value in the glioblastoma setting still needs to be evaluated.

In humans, the blood–brain barrier (BBB) plays a significant role in controlling which cells and drugs enter the brain, and there may be interspecies differences in the BBB in humans and rodents. In addition, while the BBB may be leaky in central parts of human glioblastomas, there are often pronounced and widespread infiltration of the brain parenchyma, where an intact BBB is supposed to be present [180].

Another critical issue is that even though surgical resection is first-line treatment in glioblastomas, novel therapies are rarely investigated in this context in preclinical models. For TAM-targeting therapies, this is highly relevant, since surgical resection may increase the number of TAMs in recurrent glioblastomas [158].

To get a better correlation between animal models and human glioblastoma, more studies investigating human glioblastomas and their TME are essential to get a better understanding of the complexity of these tumors. This is possible today, with all the new tools that allow the investigation of gene expression in single cells and the spatial relationship between cells.

## 5. Single-Cell Omics in Characterization of the Glioblastoma Microenvironment

With recent technological advances in single-cell and spatial sequencing, we are now able to characterize glioblastoma tumors profoundly. The extent of our ability to extract data from glioblastomas has been tested in a recent study, which comprehensively characterized 99 tumors with integrated analysis of single-cell mRNA expression, as well as tumor proteomic, genomic, post-translational modification, and metabolomic data [181]. These methods, which generate large amounts of data with few samples, can be utilized to develop ideas for new therapeutic strategies or to evaluate the effects of TAM-targeting treatments. Using single-cell RNA- or spatial-seq technologies, we can identify changes in cellular or spatial expression patterns and how this potentially affects, e.g., T-cell exhaustion, glioma proliferation, or other markers. Future clinical studies should not only investigate whether drugs targeting the glioblastoma TME are effective, but also elucidate how the drugs affect the TME, comparing naïve and treated tumors in order to potentially explain why treatments are effective or not.

As costs decrease for these technology platforms, the remaining challenge lies mainly in analyzing the enormous amounts of data and deriving information that will benefit patients. These challenges are met by research labs, who produce comprehensible tools that help scientists in analyzing their data through methods—such as principal component analysis, differential gene expression analysis, survival analysis, and unsupervised clustering techniques—to explore and extract biological insights. An example is the user-friendly Seurat [182]; an R-package for the analysis of single-cell data that enables scientists specialized in biology to explore larger datasets, which have previously required a certain level of programming knowledge. Increased simplification and accessibility have been introduced by different companies developing simple, user-friendly platforms for single-cell gene expression studies. These include a full package of, e.g., a droplet-based microfluidics system to capture and barcode single cells in droplets, simple kits for sequencing library preparation, and analysis software to easily process, analyze and visualize the data [183].

Although many studies have characterized glioblastoma cancer cells [34,38,184], only few studies have investigated and characterized the immune microenvironment. A recent study investigated single-cell mRNA expression profiles in a murine glioma model and found multiple profiles for both microglia and macrophages, with expression patterns indicating functional diversity [32], while another study investigated ligand–receptor interactions in human single-cell data and found TAMs to express genes involved in cancer cell invasion and angiogenesis [185]. These studies strongly indicate that TAMs cannot be grouped as one subpopulation, but different phenotypes may present themselves as being specifically critical in tumor progression and patient survival with distinct cellular markers.

As most studies investigating single-cell mRNA expression have been limited to data that lack spatial information, the emergence of multiple techniques that preserve cells’ spatial location adds an extra dimension of knowledge [186]. These techniques support research aiming to elucidate the function of TAMs in the TME, and how they affect their neighboring cells.

In recent years, studies have provided an exceptionally in-depth characterization of treatment-naïve glioblastomas [38,181,184], and future studies will likely characterize the effects of newer therapies and guide us in optimizing treatment strategies. These advances may pinpoint treatments that impact human tumors and enhance our understanding of cellular interactions for proposing treatment combinations.

## 6. Conclusions and Perspectives

The therapeutic targeting of TAMs in glioblastoma has yet to improve patient outcomes. This may be due to our incomplete understanding of the heterogeneity of TAMs and which subpopulations aid tumor growth, but also the insufficiency of TAM targeting as monotherapy.

It is clear that TAMs interact with cancer cells and play a significant role in tumor progression, although our understanding is incomplete. There is a need to dissect the phenotypically and functionally heterogeneous population of TAMs and map their functional roles across tumor anatomic structures. We expect that the near future will bring about several studies elucidating TAM phenotypes and functions using novel single-cell omics and spatial techniques.

A high number of clinical trials targeting TAMs as monotherapies and as combination therapies are currently ongoing and results are pending. These, in combination with more knowledge of TAM biology, might redefine glioblastoma treatment in the future.

## Figures and Tables

**Table 1:** Overview of selected clinical and experimental strategies targeting TAMs.

Targeting Strategy	Target	Clinical/Experimental	Result	Reference
**TAM recruitment**	CCL2-CCR2 axis	Clinical phase 1/2 trials with anti-CCL2 mAb or CCR2 small molecule inhibitor in different solid tumors	Carlumab, anti-CCL2 mAb: well-tolerated, but no durable anti-tumor effect PF-04136309, CCR2 small molecule inhibitor: well tolerated, some anti-tumor effect	[132,133] [134]
	CXCL12-CXCR4 axis	Clinical phase 1/2 trials with CXCR4 antagonist in glioblastoma and high grade gliomas	Plerixafor, CXCR4 antagonist: - Glioblastoma patients treated with concurrent chemoradiation. Treatment well-tolerated and improved median survival (21.3 months) - Follow-up trial of NCT01977677 with whole brain radiation. Results are pending - High grade glioma (mostly glioblastoma) patients treated with Plerixafor in combination with Bevacizumab. Treatment well-tolerated, but no effect on survival	NCT01977677 [135] NCT03746080 [136]
**Reprogramming TAM polarization and increasing** **anti-tumor TAMs**	SIRPα-CD47 pathway	Clinical phase 1 trial with anti-CD47 mAb in solid tumors	Hu5F9-G4 mAb: well tolerated, but final results are pending	[137]
	TLRs	Clinical phase 1/2 trials with TLR agonist small molecule inhibitors in solid cancers and lymphomas	Several TLR agonists are tested: there is limited effects in general, except the exception below Imiquimod: well-tolerated, and partial responses observed in skin/chest wall metastases in breast cancer patients In glioblastoma, TLR agonists are tested as immune adjuvants for immunotherapy: results are pending	[138] [139] NCT03392545 NCT01204684
	CD40	Clinical phase 1 trials in pediatric CNS tumors, solid tumors and gliomas with anti-CD40 antibodies	Anti-CD40 mAb in pediatric CNS tumors: results are pending Anti-CD40 mAb in combination with anti-PD-L1 and D2C7-IT in recurrent glioma: results are pending Anti-CD40 mAb in combination with CSF1R inhibitor: results are pending	NCT03389802 NCT04547777 NCT02760797
	NF-kB	Experimental mouse models of glioblastoma, prostate and pancreatic cancer	NF-kB inhibition in TAMs leads to M1 polarization	[140,141,142]
**Decreasing tumor-promoting TAMs**	CSF1R	Numerous clinical trials in solid and hematological tumors testing small molecule inhibitors and monoclonal antibodies Experimental mouse models of glioblastoma	PLX3397 small molecule inhibitor: - FDA approval of for tynosynovial giant cell tumor - Phase 2 trial: well tolerated, but no anti-tumor effect in glioblastoma - Phase 1/2 trial in glioblastoma, in combination with radio- and chemotherapy: results are pending BLZ945 small molecule inhibitor: - Phase 1/2 trial in advanced solid tumors in combination with anti-PD-1: results are pending - M2 polarization in glioblastoma mouse models - Improves the effect of radiation therapy by delaying or preventing recurrence	[143] [144] NCT01790503 NCT02829723 [145] [129, 146]
	PD-1/PD-L1	Clinical trial with anti-PD-1 in recurrent glioblastoma Clinical phase 3 trial with anti-PD-1 in recurrent glioblastoma Clinical phase 3 trials with anti-PD-1 in newly diagnosed glioblastoma	Improved survival with neoadjuvant pembrolizumab treatment compared to adjuvant Comparison of Nivolumab with Bevacizumab. No difference in overall survival, but more durable responses with PD-1 blockade Nivolumab compared to TMZ/TMZ+ radiation in patients with unmethylated/methylated MGMT promoter. Results for all patients show no effect on overall survival. However, MGMT promoter methylated patients had increased survival	[147] [148] NCT02617589 NCT02667587 [149]
	CD73	Experimental glioblastoma model with CD73^-/-^ mice Clinical phase 1 and 2 trials in solid tumors	Significant improved survival of CD73^-/-^ mice after anti-PD-1 and anti-CTLA-4 treatment compared to untreated mice as well as treated wildtype mice CD73 inhibition is tested alone or in combination with immune checkpoint inhibitors: results are pending	[150] [151]
	CD39	Experimental glioma mouse model and TCGA glioblastoma patient data Clinical phase 1 trial in advanced solid tumors	CD39 expression in TAMs associates with glioma grade and contributes to T cell dysfunction Anti-CD39 antibody (SRF617): results are pending	[152] NCT04336098
	CD163	Experimental melanoma mouse model Experimental in vitro set-up with human glioma-derived cells	Depletion of CD163^+^ (M2-like) TAM population leads to tumor regression Human-derived CD163^+^ TAMs inhibit effector T cells	[153] [66]
	CD204	TCGA, CGGA, and glioma patient cohorts	CD204 expression is associated with survival and correlates with malignancy grade in gliomas	[7,154]

Abbreviations: monoclonal antibody (mAb), The Cancer Genome Atlas (TCGA), Chinese Glioma Genome Atlas (CGGA).
